# 1,4-Diferrocenylbutane-1,4-dione

**DOI:** 10.1107/S1600536808003218

**Published:** 2008-02-06

**Authors:** Mustafa Tombul, Adnan Bulut, Kutalmış Güven, Orhan Büyükgüngör

**Affiliations:** a Department of Chemistry, Faculty of Arts and Science, University of Kırıkkale, Campus, Yahşihan, 71450 Kırıkkale, Turkey; b Department of Physics, Faculty of Arts and Science, University of Kırıkkale, Campus, Yahşihan, 71450 Kırıkkale, Turkey; c Department of Physics, Faculty of Arts and Science, Ondokuz Mayıs University, 55139 Samsun, Turkey

## Abstract

In the crystal structure of the title compound, [Fe_2_(C_5_H_5_)_2_(C_14_H_12_O_2_)], each carbonyl group is coplanar with the adjacent cyclo­penta­dienyl ring, thus maximizing the π-orbital overlap and electronic inter­actions between the groups. In the crystal structure, there are inter- and intra­molecular C—H⋯O contacts.

## Related literature

For related literature, see: Brown *et al.* (2005 ▶); Chidsey *et al.* (1990 ▶); Creager & Rowe (1997 ▶); Gemici (2005 ▶); Hickman *et al.* (1991 ▶); Kealy & Pauson (1951 ▶); Miller *et al.* (1988 ▶); Navarro *et al.* (2005 ▶); Nicolosi *et al.* (1994 ▶); Okochi *et al.* (2005 ▶); Pugh *et al.* (2006 ▶); Sawamura & Ito (1992 ▶); Togni & Hayashi (1995 ▶). 
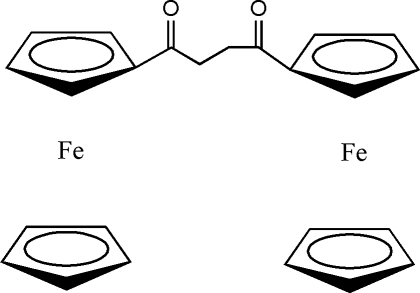


## Experimental

### 

#### Crystal data

[Fe_2_(C_5_H_5_)_2_(C_14_H_12_O_2_)]
*M* *_r_* = 454.12
Orthorhombic, 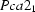

*a* = 10.4175 (7) Å
*b* = 18.5954 (10) Å
*c* = 9.9690 (6) Å
*V* = 1931.2 (2) Å^3^
*Z* = 4
Mo *K*α radiation
μ = 1.52 mm^−1^
*T* = 298 (2) K
0.45 × 0.33 × 0.08 mm

#### Data collection

Stoe IPDS2 diffractometer
Absorption correction: integration (*X-RED32*; Stoe & Cie, 2002 ▶) *T*_min_ = 0.525, *T*_max_ = 0.899
11622 measured reflections
3612 independent reflections
2941 reflections with *I* > 2σ(*I*)
*R*_int_ = 0.0383

#### Refinement

*R*[*F* ^2^ > 2σ(*F* ^2^)] = 0.036
*wR*(*F* ^2^) = 0.075
*S* = 1.01
3612 reflections
254 parameters
1 restraint
H-atom parameters not refined
Δρ_max_ = 0.36 e Å^−3^
Δρ_min_ = −0.22 e Å^−3^
Absolute structure: Flack (1983 ▶), 1418 Freidel pairs
Flack parameter: 0.01 (2)

### 

Data collection: *X-AREA* (Stoe & Cie, 2002 ▶); cell refinement: *X-AREA*; data reduction: *X-RED32* (Stoe & Cie, 2002 ▶); program(s) used to solve structure: *SHELXS97* (Sheldrick, 2008 ▶); program(s) used to refine structure: *SHELXL97* (Sheldrick, 2008 ▶); molecular graphics: *ORTEP-3 for Windows* (Farrugia, 1997 ▶); software used to prepare material for publication: *publCIF* (Westrip, 2008 ▶).

## Supplementary Material

Crystal structure: contains datablocks global, I. DOI: 10.1107/S1600536808003218/at2537sup1.cif

Structure factors: contains datablocks I. DOI: 10.1107/S1600536808003218/at2537Isup2.hkl

Additional supplementary materials:  crystallographic information; 3D view; checkCIF report

## Figures and Tables

**Table 1:** Hydrogen-bond geometry (Å, °)

*D*—H⋯*A*	*D*—H	H⋯*A*	*D*⋯*A*	*D*—H⋯*A*
C12—H12*B*⋯O1^i^	0.97	2.51	3.448 (5)	164
C13—H13*B*⋯O2^i^	0.97	2.55	3.400 (6)	147
C23—H23⋯O1	0.93	2.60	3.499 (5)	164
C10—H10⋯O2	0.93	2.58	3.457 (6)	157

Symmetry code: (i) 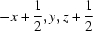.

**Fig. 1.**
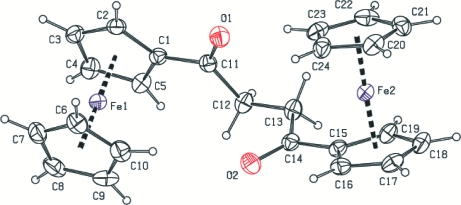
Showing the atom-labelling scheme of (I). Displacement ellipsoids are drawn at the 30% probability level.

**Fig. 2.**
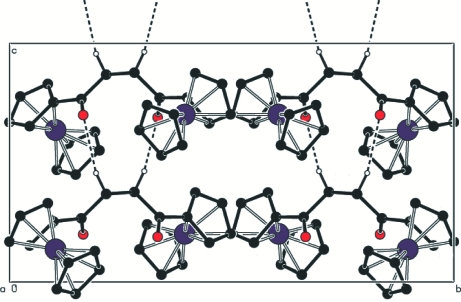
The packing diagram and the intermolecular C—H···O hydrogen bonding interactions of (I), viewed down the a axis. For clarity, H atoms not involved in hydrogen bonding have been omitted.

## supplementary crystallographic information

### Comment

Since its first discovery in 1951 (Kealy & Pauson, 1951), particular attention has been paid to ferrocene and its derivatives owing to their unique structural, spectroscopic and electrochemical properties. In recent years, a number of extensive studies has been conducted on ferrocene containing compounds and polymers, and particularly compounds exhibiting multiple ferrocene groups with the opportunity for producing mixed-valent states due to their potential use in chemical and biochemical sensors (Navarro *et al.*, 2005; Brown *et al.*, 2005; Okochi *et al.*, 2005; Hickman *et al.*, 1991), as redox active catalysts (Togni & Hayashi, 1995; Sawamura & Ito, 1992; Nicolosi *et al.*, 1994), because of their application in molecular magnets (Miller *et al.*, 1988), and for use in self-assembled monolayer chemistry (Chidsey *et al.*, 1990; Creager & Rowe, 1997). Such monolayers containing covalently connected ferrocene groups have been reported to be prepared in mixed-valent, fully oxidized or fully reduced states (Pugh *et al.*, 2006). Hence, in order to increase their potential for the aforementioned applications, the chemical properties of the monolayers can be controlled *via* application of the appropriate voltage. Due to the electron transfer of ferrocene being generally chemically and electrochemically reversible and occurring *via* an outer-sphere redox mechanism, ferrocene derivatives are not only perfect candidates to be incorporated into technological devices, but also ideal molecules for the study of interfacial electron and charge transfer. We report here the single-crystal structure of the title compound (I).

The molecular structure of (I), is shown in Fig. 1. Succinylferrocene crystallizes in the orthorhombic space group Pca2_1_ with one molecule in the asymmetric unit. The ferrocene groups themselves are in the eclipsed conformation and the bond lengths and angles associated with the ferrocene groups are as expected. Two ferrocenyl groups are almost *trans* to each other. The average values of the C—*Cg*1—*Cg*2—C and C—*Cg*3—*Cg*4—C (*Cg*1, *Cg*2, *Cg*3 and *Cg*4 are the centroids of the C1—C5, C6—C10, C15—C19 and C20—C24 rings, respectively) pseuodo-torsion angles are 5.50 (2)° and 7.13 (2)° respectively. The Fe1–*Cg*1, Fe1–*Cg*2, Fe2–*Cg*3 and Fe2–*Cg*4 distances are 1.635 (2) Å, 1.649 (2) Å, 1.645 (3)Å and 1.647 (2) Å, respectively, with *Cg*1–Fe1–*Cg*2 and *Cg*3–Fe2–*Cg*4 angles of 179.54 (10)° and 178.65 (11)°. Within the molecule, the carbonyl substituents are almost coplanar with the plane of the adjacent Cp rings (r.m.s. deviations = 0.0278 (2) Å and 0.0559 (2) Å; Cp *versus*. C=O dihedral angles: 7.42 (6)° and 13.74 (5)° respectively), therefore maximizing the π-orbital overlap and electronic interactions between the groups. In (I), the intermolecular bond lengths and angles are unexceptional; the central C—C distance, 1.492 (6) Å, is close to the value found in 1,6-diferrocenylhexane-1,6-dione, 1.506 (5) Å (Pugh *et al.*, 2006). The space between the ferrocene units is nearly close packed and the ferrocene moieties are closer to each other in the solid state (7.903 (21) Å which is the distance between Fe1 and Fe2) than they would be on average in solution.

In the crystal structure, weak inter and intramolecular C—H···O hydrogen bonding interactions link molecules (Table 1, Fig. 2) and may be effective in the stabilization of the structure.

### Experimental

The title compound (I) was prepared by employing Alkyl Lewis acid, EtAlCl_2_ (Gemici, 2005). To a CH_2_Cl_2_ solution (10 ml) containing ferrocene (1.27 g, 6.8 mmol), EtAlCl_2_ (6.8 ml; 1.0 *M* in hexane) was added dropwise at 273 K under nitrogen. The resulting mixture was stirred at 273 K for 1,5 h. The solution was then extracted with portions of CH_2_Cl_2_ (3 times; in total 75 ml), dried over Na_2_SO_4_ and evaporated to dryness. Final purification was achieved by flash chromatography on silica gel utilizing CHCl_3_ as the eluant. The product was obtained in 76% yield. ^1^H-NMR (400 MHz, CHCl_3_, δ, p.p.m.): 3.05 (broad m, 4H, COCH_2_CH_2_CO), 4.20 (s, 10H, 2xC_5_H_5_), 4.40 (broad s, 4H, C_5_H_4_), 4.76 (broad s, 4H, C_5_H_4_). X-ray quality single crystals of (I) were prepared *via* solvent evaporation from hexane/ethyl acetate solutions to give reddish platelets.

### Refinement

The H atoms were all located in a difference map, but they were repositioned geometrically. The H atoms were initially refined with soft restraints on the bond lengths and angles to regularize their geometry (C—H = 0.93 and 0.97 Å) and *U*_iso_(H) = 1.2 times *U*_eq_ of the parent atom, after which the positions were refined with riding constraints.

### Crystal data

**Table:**

[Fe_2_(C_5_H_5_)_2_(C_14_H_12_O_2_)]	*F*_000_ = 936
*M**_r_* = 454.12	*D*_x_ = 1.562 Mg m^−^^3^
Orthorhombic, *P**c**a*2_1_	Mo *K*α radiation λ = 0.71073 Å
Hall symbol: P 2c -2ac	Cell parameters from 17802 reflections
*a* = 10.4175 (7) Å	θ = 2.0–27.3º
*b* = 18.5954 (10) Å	µ = 1.52 mm^−^^1^
*c* = 9.9690 (6) Å	*T* = 298 (2) K
*V* = 1931.2 (2) Å^3^	Prismatic stick, red
*Z* = 4	0.45 × 0.33 × 0.08 mm

### Data collection

**Table:**

Stoe IPDS 2 diffractometer	3612 independent reflections
Monochromator: plane graphite	2941 reflections with *I* > 2u(*I*)
Detector resolution: 6.67 pixels mm^-1^	*R*_int_ = 0.038
*T* = 298(2) K	θ_max_ = 26.8º
ω scans	θ_min_ = 2.0º
Absorption correction: integration(X-RED32; Stoe & Cie, 2002)	*h* = −13→13
*T*_min_ = 0.525, *T*_max_ = 0.899	*k* = −20→23
11622 measured reflections	*l* = −12→12

### Refinement

**Table:**

Refinement on *F*^2^	Hydrogen site location: inferred from neighbouring sites
Least-squares matrix: full	H-atom parameters not refined
*R*[*F*^2^ > 2σ(*F*^2^)] = 0.036	*w* = 1/[σ^2^(*F*_o_^2^) + (0.0398*P*)^2^] where *P* = (*F*_o_^2^ + 2*F*_c_^2^)/3
*wR*(*F*^2^) = 0.075	(Δ/σ)_max_ = 0.001
*S* = 1.01	Δρ_max_ = 0.37 e Å^−^^3^
3612 reflections	Δρ_min_ = −0.22 e Å^−^^3^
254 parameters	Extinction correction: none
1 restraint	Absolute structure: Flack (1983), 1418 Freidel pairs
Primary atom site location: structure-invariant direct methods	Flack parameter: 0.01 (2)
Secondary atom site location: difference Fourier map	

### Special details

**Table:**

Geometry. All e.s.d.'s (except the e.s.d. in the dihedral angle between two l.s. planes) are estimated using the full covariance matrix. The cell e.s.d.'s are taken into account individually in the estimation of e.s.d.'s in distances, angles and torsion angles; correlations between e.s.d.'s in cell parameters are only used when they are defined by crystal symmetry. An approximate (isotropic) treatment of cell e.s.d.'s is used for estimating e.s.d.'s involving l.s. planes.
Refinement. Refinement of *F*^2^ against ALL reflections. The weighted *R*-factor *wR* and goodness of fit *S* are based on *F*^2^, conventional *R*-factors *R* are based on *F*, with *F* set to zero for negative *F*^2^. The threshold expression of *F*^2^ > σ(*F*^2^) is used only for calculating *R*-factors(gt) *etc*. and is not relevant to the choice of reflections for refinement. *R*-factors based on *F*^2^ are statistically about twice as large as those based on *F*, and *R*- factors based on ALL data will be even larger.

### Fractional atomic coordinates and isotropic or equivalent isotropic displacement parameters (Å^2^)

**Table:**

	*x*	*y*	*z*	*U*_iso_*/*U*_eq_	
C1	0.3828 (4)	0.9008 (2)	0.2532 (4)	0.0488 (9)	
C2	0.3776 (3)	0.9545 (2)	0.1526 (5)	0.0557 (10)	
H2	0.3145	0.9590	0.0871	0.067*	
C3	0.4842 (4)	1.0000 (2)	0.1689 (5)	0.0601 (13)	
H3	0.5037	1.0398	0.1161	0.072*	
C4	0.5553 (4)	0.9756 (2)	0.2773 (5)	0.0642 (11)	
H4	0.6302	0.9965	0.3100	0.077*	
C5	0.4951 (4)	0.9137 (2)	0.3299 (5)	0.0553 (10)	
H5	0.5239	0.8863	0.4019	0.066*	
C6	0.5458 (4)	0.8510 (3)	−0.0499 (5)	0.0676 (12)	
H6	0.4850	0.8567	−0.1174	0.081*	
C7	0.6536 (5)	0.8933 (3)	−0.0304 (6)	0.0758 (14)	
H7	0.6769	0.9327	−0.0826	0.091*	
C8	0.7222 (4)	0.8674 (3)	0.0808 (6)	0.0798 (17)	
H8	0.7980	0.8862	0.1155	0.096*	
C9	0.6542 (4)	0.8078 (3)	0.1296 (7)	0.0794 (14)	
H9	0.6783	0.7796	0.2025	0.095*	
C10	0.5444 (4)	0.7974 (3)	0.0512 (5)	0.0696 (13)	
H10	0.4822	0.7621	0.0631	0.083*	
C11	0.2960 (4)	0.8382 (2)	0.2666 (4)	0.0505 (9)	
C12	0.3305 (4)	0.7823 (2)	0.3686 (4)	0.0568 (10)	
H12A	0.4163	0.7651	0.3490	0.068*	
H12B	0.3332	0.8051	0.4561	0.068*	
C13	0.2431 (5)	0.7189 (2)	0.3771 (4)	0.0567 (9)	
H13A	0.1553	0.7359	0.3716	0.068*	
H13B	0.2540	0.6967	0.4644	0.068*	
C14	0.2621 (4)	0.6621 (2)	0.2717 (4)	0.0517 (9)	
C15	0.1743 (4)	0.5999 (2)	0.2724 (4)	0.0502 (9)	
C16	0.1563 (4)	0.5503 (2)	0.1648 (4)	0.0587 (12)	
H16	0.2017	0.5496	0.0844	0.070*	
C17	0.0573 (4)	0.5024 (2)	0.2023 (5)	0.0656 (11)	
H17	0.0262	0.4646	0.1506	0.079*	
C18	0.0139 (4)	0.5214 (2)	0.3300 (5)	0.0662 (12)	
H18	−0.0507	0.4983	0.3777	0.079*	
C19	0.0845 (4)	0.5817 (2)	0.3745 (4)	0.0571 (10)	
H19	0.0742	0.6052	0.4561	0.069*	
C20	−0.1607 (5)	0.6109 (3)	0.0730 (5)	0.0652 (13)	
H20	−0.1936	0.5729	0.0231	0.078*	
C21	−0.1998 (4)	0.6299 (3)	0.2023 (6)	0.0654 (11)	
H21	−0.2628	0.6072	0.2532	0.078*	
C22	−0.1253 (4)	0.6901 (3)	0.2414 (6)	0.0710 (15)	
H22	−0.1307	0.7140	0.3232	0.085*	
C23	−0.0425 (4)	0.7074 (2)	0.1362 (6)	0.0691 (12)	
H23	0.0167	0.7449	0.1355	0.083*	
C24	−0.0647 (4)	0.6579 (3)	0.0306 (5)	0.0671 (12)	
H24	−0.0228	0.6569	−0.0518	0.081*	
O1	0.1985 (2)	0.83401 (17)	0.1994 (3)	0.0669 (7)	
O2	0.3494 (2)	0.66641 (17)	0.1891 (3)	0.0680 (8)	
Fe1	0.54062 (5)	0.89622 (3)	0.13663 (6)	0.04646 (14)	
Fe2	−0.00932 (5)	0.60553 (3)	0.20155 (6)	0.04697 (13)	

### Atomic displacement parameters (Å^2^)

**Table:**

	*U*^11^	*U*^22^	*U*^33^	*U*^12^	*U*^13^	*U*^23^
C1	0.052 (2)	0.047 (2)	0.047 (2)	0.0039 (18)	0.0009 (17)	−0.002 (2)
C2	0.054 (2)	0.058 (3)	0.055 (3)	0.0143 (17)	0.0015 (18)	0.004 (2)
C3	0.071 (2)	0.049 (2)	0.060 (4)	0.0085 (18)	0.0098 (19)	0.003 (2)
C4	0.075 (3)	0.053 (3)	0.064 (3)	−0.012 (2)	−0.002 (2)	−0.009 (2)
C5	0.070 (2)	0.050 (2)	0.046 (2)	−0.0058 (19)	−0.0101 (18)	−0.0018 (19)
C6	0.068 (3)	0.077 (3)	0.057 (3)	−0.001 (2)	0.007 (2)	−0.015 (3)
C7	0.070 (3)	0.072 (3)	0.085 (4)	−0.004 (3)	0.031 (3)	−0.004 (3)
C8	0.044 (2)	0.081 (4)	0.114 (5)	−0.001 (2)	0.008 (2)	−0.023 (3)
C9	0.072 (3)	0.069 (3)	0.097 (4)	0.032 (2)	−0.010 (3)	−0.011 (4)
C10	0.071 (3)	0.063 (3)	0.074 (3)	−0.005 (2)	0.011 (2)	−0.020 (3)
C11	0.055 (2)	0.056 (2)	0.0403 (19)	0.0052 (18)	0.0055 (18)	−0.002 (2)
C12	0.074 (3)	0.055 (3)	0.042 (2)	−0.007 (2)	−0.0032 (17)	0.000 (2)
C13	0.075 (2)	0.059 (2)	0.0362 (19)	−0.007 (2)	0.0026 (16)	−0.001 (2)
C14	0.058 (2)	0.055 (2)	0.042 (2)	0.0067 (18)	−0.0024 (18)	0.008 (2)
C15	0.061 (2)	0.047 (2)	0.043 (2)	0.0057 (18)	−0.0036 (18)	0.006 (2)
C16	0.065 (2)	0.053 (2)	0.058 (4)	0.0120 (17)	0.0003 (17)	−0.007 (2)
C17	0.084 (3)	0.042 (2)	0.071 (3)	0.0021 (18)	−0.015 (3)	−0.003 (3)
C18	0.077 (3)	0.057 (3)	0.065 (3)	−0.011 (2)	−0.010 (2)	0.019 (2)
C19	0.075 (3)	0.052 (2)	0.044 (2)	−0.003 (2)	−0.0067 (19)	0.008 (2)
C20	0.077 (3)	0.056 (3)	0.063 (3)	−0.007 (2)	−0.020 (2)	0.002 (3)
C21	0.050 (2)	0.070 (3)	0.076 (3)	−0.0053 (18)	0.000 (3)	−0.001 (3)
C22	0.062 (2)	0.060 (3)	0.091 (4)	0.017 (2)	−0.015 (2)	−0.019 (3)
C23	0.063 (2)	0.045 (2)	0.099 (3)	−0.0026 (19)	−0.030 (3)	0.011 (3)
C24	0.062 (2)	0.073 (3)	0.066 (3)	−0.008 (2)	−0.009 (2)	0.022 (3)
O1	0.0594 (15)	0.080 (2)	0.0610 (16)	−0.0037 (13)	−0.0077 (16)	0.0057 (19)
O2	0.0693 (16)	0.078 (2)	0.0571 (18)	0.0002 (14)	0.0090 (15)	−0.0070 (17)
Fe1	0.0457 (3)	0.0446 (3)	0.0491 (3)	0.0020 (2)	−0.0029 (3)	−0.0021 (4)
Fe2	0.0525 (3)	0.0418 (3)	0.0466 (3)	−0.0013 (2)	−0.0022 (2)	0.0020 (4)

### Geometric parameters (Å, °)

**Table:**

C1—C2	1.417 (6)	C13—C14	1.503 (6)
C1—C5	1.418 (6)	C13—H13A	0.9700
C1—C11	1.480 (6)	C13—H13B	0.9700
C1—Fe1	2.015 (4)	C14—O2	1.229 (4)
C2—C3	1.406 (6)	C14—C15	1.475 (6)
C2—Fe1	2.022 (3)	C15—C19	1.423 (6)
C2—H2	0.9300	C15—C16	1.427 (6)
C3—C4	1.387 (6)	C15—Fe2	2.042 (4)
C3—Fe1	2.044 (4)	C16—C17	1.413 (6)
C3—H3	0.9300	C16—Fe2	2.042 (4)
C4—C5	1.411 (6)	C16—H16	0.9300
C4—Fe1	2.042 (4)	C17—C18	1.396 (7)
C4—H4	0.9300	C17—Fe2	2.039 (4)
C5—Fe1	2.011 (5)	C17—H17	0.9300
C5—H5	0.9300	C18—C19	1.412 (6)
C6—C7	1.386 (7)	C18—Fe2	2.035 (4)
C6—C10	1.417 (7)	C18—H18	0.9300
C6—Fe1	2.041 (5)	C19—Fe2	2.032 (4)
C6—H6	0.9300	C19—H19	0.9300
C7—C8	1.404 (8)	C20—C24	1.394 (6)
C7—Fe1	2.040 (5)	C20—C21	1.397 (7)
C7—H7	0.9300	C20—Fe2	2.034 (5)
C8—C9	1.404 (7)	C20—H20	0.9300
C8—Fe1	2.043 (4)	C21—C22	1.417 (6)
C8—H8	0.9300	C21—Fe2	2.035 (4)
C9—C10	1.399 (7)	C21—H21	0.9300
C9—Fe1	2.027 (4)	C22—C23	1.396 (7)
C9—H9	0.9300	C22—Fe2	2.023 (4)
C10—Fe1	2.026 (5)	C22—H22	0.9300
C10—H10	0.9300	C23—C24	1.418 (7)
C11—O1	1.219 (5)	C23—Fe2	2.033 (4)
C11—C12	1.499 (6)	C23—H23	0.9300
C12—C13	1.492 (6)	C24—Fe2	2.046 (4)
C12—H12A	0.9700	C24—H24	0.9300
C12—H12B	0.9700		
			
C2—C1—C5	107.1 (4)	C20—C21—C22	107.1 (5)
C2—C1—C11	126.5 (4)	C20—C21—Fe2	69.9 (3)
C5—C1—C11	126.0 (4)	C22—C21—Fe2	69.1 (2)
C2—C1—Fe1	69.7 (2)	C20—C21—H21	126.5
C5—C1—Fe1	69.2 (2)	C22—C21—H21	126.5
C11—C1—Fe1	121.2 (3)	Fe2—C21—H21	126.1
C3—C2—C1	108.2 (4)	C23—C22—C21	108.3 (5)
C3—C2—Fe1	70.6 (2)	C23—C22—Fe2	70.3 (2)
C1—C2—Fe1	69.2 (2)	C21—C22—Fe2	70.0 (3)
C3—C2—H2	125.9	C23—C22—H22	125.8
C1—C2—H2	125.9	C21—C22—H22	125.8
Fe1—C2—H2	125.9	Fe2—C22—H22	125.4
C4—C3—C2	108.4 (4)	C22—C23—C24	107.9 (4)
C4—C3—Fe1	70.1 (2)	C22—C23—Fe2	69.5 (3)
C2—C3—Fe1	68.9 (2)	C24—C23—Fe2	70.1 (2)
C4—C3—H3	125.8	C22—C23—H23	126.0
C2—C3—H3	125.8	C24—C23—H23	126.0
Fe1—C3—H3	126.7	Fe2—C23—H23	125.9
C3—C4—C5	108.6 (4)	C20—C24—C23	107.4 (5)
C3—C4—Fe1	70.2 (3)	C20—C24—Fe2	69.6 (3)
C5—C4—Fe1	68.4 (2)	C23—C24—Fe2	69.2 (3)
C3—C4—H4	125.7	C20—C24—H24	126.3
C5—C4—H4	125.7	C23—C24—H24	126.3
Fe1—C4—H4	127.2	Fe2—C24—H24	126.5
C4—C5—C1	107.7 (4)	C5—Fe1—C1	41.26 (16)
C4—C5—Fe1	70.8 (3)	C5—Fe1—C2	68.88 (18)
C1—C5—Fe1	69.5 (2)	C1—Fe1—C2	41.09 (17)
C4—C5—H5	126.1	C5—Fe1—C10	123.65 (19)
C1—C5—H5	126.1	C1—Fe1—C10	107.25 (18)
Fe1—C5—H5	125.1	C2—Fe1—C10	122.41 (18)
C7—C6—C10	108.0 (4)	C5—Fe1—C9	107.6 (2)
C7—C6—Fe1	70.1 (3)	C1—Fe1—C9	122.0 (2)
C10—C6—Fe1	69.0 (3)	C2—Fe1—C9	158.17 (19)
C7—C6—H6	126.0	C10—Fe1—C9	40.4 (2)
C10—C6—H6	126.0	C5—Fe1—C7	157.3 (2)
Fe1—C6—H6	126.5	C1—Fe1—C7	160.5 (2)
C6—C7—C8	109.2 (5)	C2—Fe1—C7	124.3 (2)
C6—C7—Fe1	70.2 (3)	C10—Fe1—C7	67.8 (2)
C8—C7—Fe1	70.0 (3)	C9—Fe1—C7	67.3 (2)
C6—C7—H7	125.4	C5—Fe1—C6	161.01 (18)
C8—C7—H7	125.4	C1—Fe1—C6	124.33 (18)
Fe1—C7—H7	125.9	C2—Fe1—C6	108.36 (19)
C9—C8—C7	106.7 (5)	C10—Fe1—C6	40.8 (2)
C9—C8—Fe1	69.2 (2)	C9—Fe1—C6	67.6 (2)
C7—C8—Fe1	69.8 (3)	C7—Fe1—C6	39.70 (19)
C9—C8—H8	126.6	C5—Fe1—C4	40.76 (17)
C7—C8—H8	126.6	C1—Fe1—C4	68.56 (17)
Fe1—C8—H8	125.9	C2—Fe1—C4	67.73 (19)
C10—C9—C8	109.1 (5)	C10—Fe1—C4	160.6 (2)
C10—C9—Fe1	69.7 (2)	C9—Fe1—C4	124.5 (2)
C8—C9—Fe1	70.4 (3)	C7—Fe1—C4	122.4 (2)
C10—C9—H9	125.4	C6—Fe1—C4	157.16 (19)
C8—C9—H9	125.4	C5—Fe1—C8	121.4 (2)
Fe1—C9—H9	126.0	C1—Fe1—C8	157.5 (2)
C9—C10—C6	107.0 (4)	C2—Fe1—C8	160.0 (2)
C9—C10—Fe1	69.9 (3)	C10—Fe1—C8	68.29 (19)
C6—C10—Fe1	70.2 (3)	C9—Fe1—C8	40.3 (2)
C9—C10—H10	126.5	C7—Fe1—C8	40.2 (2)
C6—C10—H10	126.5	C6—Fe1—C8	67.6 (2)
Fe1—C10—H10	125.0	C4—Fe1—C8	107.9 (2)
O1—C11—C1	120.7 (4)	C5—Fe1—C3	68.19 (18)
O1—C11—C12	121.9 (4)	C1—Fe1—C3	68.56 (17)
C1—C11—C12	117.4 (3)	C2—Fe1—C3	40.46 (16)
C13—C12—C11	116.2 (4)	C10—Fe1—C3	158.24 (19)
C13—C12—H12A	108.2	C9—Fe1—C3	160.00 (18)
C11—C12—H12A	108.2	C7—Fe1—C3	108.6 (2)
C13—C12—H12B	108.2	C6—Fe1—C3	122.7 (2)
C11—C12—H12B	108.2	C4—Fe1—C3	39.69 (18)
H12A—C12—H12B	107.4	C8—Fe1—C3	123.8 (2)
C12—C13—C14	115.8 (4)	C22—Fe2—C19	106.9 (2)
C12—C13—H13A	108.3	C22—Fe2—C23	40.3 (2)
C14—C13—H13A	108.3	C19—Fe2—C23	123.9 (2)
C12—C13—H13B	108.3	C22—Fe2—C20	67.8 (2)
C14—C13—H13B	108.3	C19—Fe2—C20	156.70 (19)
H13A—C13—H13B	107.4	C23—Fe2—C20	67.71 (18)
O2—C14—C15	120.9 (4)	C22—Fe2—C21	40.87 (18)
O2—C14—C13	121.3 (4)	C19—Fe2—C21	121.0 (2)
C15—C14—C13	117.8 (4)	C23—Fe2—C21	68.17 (19)
C19—C15—C16	107.4 (4)	C20—Fe2—C21	40.2 (2)
C19—C15—C14	126.7 (4)	C22—Fe2—C18	123.0 (2)
C16—C15—C14	125.8 (4)	C19—Fe2—C18	40.62 (17)
C19—C15—Fe2	69.2 (2)	C23—Fe2—C18	159.7 (2)
C16—C15—Fe2	69.5 (2)	C20—Fe2—C18	121.74 (19)
C14—C15—Fe2	122.6 (3)	C21—Fe2—C18	106.5 (2)
C17—C16—C15	107.7 (4)	C22—Fe2—C17	159.0 (2)
C17—C16—Fe2	69.6 (2)	C19—Fe2—C17	68.14 (19)
C15—C16—Fe2	69.5 (2)	C23—Fe2—C17	159.3 (2)
C17—C16—H16	126.2	C20—Fe2—C17	108.2 (2)
C15—C16—H16	126.2	C21—Fe2—C17	122.74 (18)
Fe2—C16—H16	126.2	C18—Fe2—C17	40.1 (2)
C18—C17—C16	108.6 (4)	C22—Fe2—C15	122.14 (18)
C18—C17—Fe2	69.8 (3)	C19—Fe2—C15	40.91 (17)
C16—C17—Fe2	69.9 (2)	C23—Fe2—C15	108.56 (16)
C18—C17—H17	125.7	C20—Fe2—C15	161.2 (2)
C16—C17—H17	125.7	C21—Fe2—C15	157.4 (2)
Fe2—C17—H17	126.2	C18—Fe2—C15	68.36 (17)
C17—C18—C19	108.6 (4)	C17—Fe2—C15	68.37 (16)
C17—C18—Fe2	70.1 (3)	C22—Fe2—C16	158.68 (19)
C19—C18—Fe2	69.5 (2)	C19—Fe2—C16	68.64 (17)
C17—C18—H18	125.7	C23—Fe2—C16	123.7 (2)
C19—C18—H18	125.7	C20—Fe2—C16	124.5 (2)
Fe2—C18—H18	126.3	C21—Fe2—C16	159.50 (19)
C18—C19—C15	107.8 (4)	C18—Fe2—C16	68.02 (19)
C18—C19—Fe2	69.8 (2)	C17—Fe2—C16	40.52 (17)
C15—C19—Fe2	69.9 (2)	C15—Fe2—C16	40.91 (16)
C18—C19—H19	126.1	C22—Fe2—C24	68.0 (2)
C15—C19—H19	126.1	C19—Fe2—C24	161.24 (18)
Fe2—C19—H19	125.7	C23—Fe2—C24	40.7 (2)
C24—C20—C21	109.3 (5)	C20—Fe2—C24	39.96 (17)
C24—C20—Fe2	70.5 (3)	C21—Fe2—C24	67.8 (2)
C21—C20—Fe2	69.9 (2)	C18—Fe2—C24	157.4 (2)
C24—C20—H20	125.3	C17—Fe2—C24	123.1 (2)
C21—C20—H20	125.3	C15—Fe2—C24	125.22 (17)
Fe2—C20—H20	125.8	C16—Fe2—C24	109.14 (19)
			
C5—C1—C2—C3	−0.8 (5)	C5—C4—Fe1—C10	−42.4 (7)
C11—C1—C2—C3	−174.5 (4)	C3—C4—Fe1—C9	163.2 (3)
Fe1—C1—C2—C3	−60.1 (3)	C5—C4—Fe1—C9	−76.3 (3)
C5—C1—C2—Fe1	59.3 (3)	C3—C4—Fe1—C7	80.0 (3)
C11—C1—C2—Fe1	−114.4 (4)	C5—C4—Fe1—C7	−159.5 (3)
C1—C2—C3—C4	0.0 (5)	C3—C4—Fe1—C6	47.1 (6)
Fe1—C2—C3—C4	−59.2 (3)	C5—C4—Fe1—C6	167.6 (4)
C1—C2—C3—Fe1	59.2 (3)	C3—C4—Fe1—C8	121.8 (3)
C2—C3—C4—C5	0.7 (5)	C5—C4—Fe1—C8	−117.7 (3)
Fe1—C3—C4—C5	−57.8 (3)	C5—C4—Fe1—C3	120.5 (4)
C2—C3—C4—Fe1	58.5 (3)	C9—C8—Fe1—C5	80.0 (4)
C3—C4—C5—C1	−1.2 (5)	C7—C8—Fe1—C5	−162.1 (3)
Fe1—C4—C5—C1	−60.0 (3)	C9—C8—Fe1—C1	45.6 (7)
C3—C4—C5—Fe1	58.9 (3)	C7—C8—Fe1—C1	163.5 (4)
C2—C1—C5—C4	1.2 (5)	C9—C8—Fe1—C2	−163.5 (6)
C11—C1—C5—C4	174.9 (4)	C7—C8—Fe1—C2	−45.6 (8)
Fe1—C1—C5—C4	60.8 (3)	C9—C8—Fe1—C10	−37.1 (4)
C2—C1—C5—Fe1	−59.7 (3)	C7—C8—Fe1—C10	80.8 (4)
C11—C1—C5—Fe1	114.1 (4)	C7—C8—Fe1—C9	117.9 (5)
C10—C6—C7—C8	0.6 (5)	C9—C8—Fe1—C7	−117.9 (5)
Fe1—C6—C7—C8	59.4 (4)	C9—C8—Fe1—C6	−81.2 (4)
C10—C6—C7—Fe1	−58.8 (3)	C7—C8—Fe1—C6	36.7 (3)
C6—C7—C8—C9	0.1 (6)	C9—C8—Fe1—C4	122.6 (4)
Fe1—C7—C8—C9	59.6 (3)	C7—C8—Fe1—C4	−119.4 (3)
C6—C7—C8—Fe1	−59.5 (3)	C9—C8—Fe1—C3	163.4 (3)
C7—C8—C9—C10	−0.8 (6)	C7—C8—Fe1—C3	−78.7 (4)
Fe1—C8—C9—C10	59.2 (3)	C4—C3—Fe1—C5	37.3 (3)
C7—C8—C9—Fe1	−60.0 (3)	C2—C3—Fe1—C5	−82.6 (3)
C8—C9—C10—C6	1.1 (6)	C4—C3—Fe1—C1	81.8 (3)
Fe1—C9—C10—C6	60.7 (3)	C2—C3—Fe1—C1	−38.1 (3)
C8—C9—C10—Fe1	−59.6 (3)	C4—C3—Fe1—C2	119.9 (4)
C7—C6—C10—C9	−1.0 (5)	C4—C3—Fe1—C10	164.7 (5)
Fe1—C6—C10—C9	−60.5 (3)	C2—C3—Fe1—C10	44.8 (6)
C7—C6—C10—Fe1	59.5 (3)	C4—C3—Fe1—C9	−44.1 (8)
C2—C1—C11—O1	−10.7 (6)	C2—C3—Fe1—C9	−164.0 (6)
C5—C1—C11—O1	176.7 (4)	C4—C3—Fe1—C7	−118.7 (3)
Fe1—C1—C11—O1	−97.5 (4)	C2—C3—Fe1—C7	121.4 (3)
C2—C1—C11—C12	171.3 (4)	C4—C3—Fe1—C6	−160.2 (3)
C5—C1—C11—C12	−1.3 (6)	C2—C3—Fe1—C6	79.8 (3)
Fe1—C1—C11—C12	84.5 (4)	C2—C3—Fe1—C4	−119.9 (4)
O1—C11—C12—C13	3.7 (6)	C4—C3—Fe1—C8	−76.8 (3)
C1—C11—C12—C13	−178.3 (4)	C2—C3—Fe1—C8	163.3 (3)
C11—C12—C13—C14	79.5 (5)	C23—C22—Fe2—C19	122.9 (3)
C12—C13—C14—O2	4.0 (6)	C21—C22—Fe2—C19	−118.1 (3)
C12—C13—C14—C15	−177.4 (4)	C21—C22—Fe2—C23	119.0 (5)
O2—C14—C15—C19	168.3 (4)	C23—C22—Fe2—C20	−81.2 (3)
C13—C14—C15—C19	−10.2 (6)	C21—C22—Fe2—C20	37.8 (3)
O2—C14—C15—C16	−17.5 (6)	C23—C22—Fe2—C21	−119.0 (5)
C13—C14—C15—C16	164.0 (4)	C23—C22—Fe2—C18	164.4 (3)
O2—C14—C15—Fe2	−104.6 (4)	C21—C22—Fe2—C18	−76.6 (4)
C13—C14—C15—Fe2	76.9 (5)	C23—C22—Fe2—C17	−164.3 (5)
C19—C15—C16—C17	−0.4 (5)	C21—C22—Fe2—C17	−45.2 (7)
C14—C15—C16—C17	−175.5 (4)	C23—C22—Fe2—C15	80.8 (3)
Fe2—C15—C16—C17	−59.4 (3)	C21—C22—Fe2—C15	−160.2 (3)
C19—C15—C16—Fe2	59.0 (3)	C23—C22—Fe2—C16	48.5 (7)
C14—C15—C16—Fe2	−116.1 (4)	C21—C22—Fe2—C16	167.5 (5)
C15—C16—C17—C18	0.1 (5)	C23—C22—Fe2—C24	−37.9 (3)
Fe2—C16—C17—C18	−59.3 (3)	C21—C22—Fe2—C24	81.1 (3)
C15—C16—C17—Fe2	59.4 (3)	C18—C19—Fe2—C22	121.4 (3)
C16—C17—C18—C19	0.3 (5)	C15—C19—Fe2—C22	−119.9 (3)
Fe2—C17—C18—C19	−59.1 (3)	C18—C19—Fe2—C23	162.2 (3)
C16—C17—C18—Fe2	59.3 (3)	C15—C19—Fe2—C23	−79.1 (3)
C17—C18—C19—C15	−0.5 (5)	C18—C19—Fe2—C20	48.4 (6)
Fe2—C18—C19—C15	−59.9 (3)	C15—C19—Fe2—C20	167.1 (5)
C17—C18—C19—Fe2	59.4 (3)	C18—C19—Fe2—C21	79.0 (3)
C16—C15—C19—C18	0.5 (5)	C15—C19—Fe2—C21	−162.2 (3)
C14—C15—C19—C18	175.6 (4)	C15—C19—Fe2—C18	118.7 (4)
Fe2—C15—C19—C18	59.8 (3)	C18—C19—Fe2—C17	−37.0 (3)
C16—C15—C19—Fe2	−59.3 (3)	C15—C19—Fe2—C17	81.7 (3)
C14—C15—C19—Fe2	115.8 (4)	C18—C19—Fe2—C15	−118.7 (4)
C24—C20—C21—C22	−0.3 (5)	C18—C19—Fe2—C16	−80.7 (3)
Fe2—C20—C21—C22	59.3 (3)	C15—C19—Fe2—C16	38.0 (2)
C24—C20—C21—Fe2	−59.7 (3)	C18—C19—Fe2—C24	−167.5 (6)
C20—C21—C22—C23	0.3 (5)	C15—C19—Fe2—C24	−48.8 (7)
Fe2—C21—C22—C23	60.1 (3)	C24—C23—Fe2—C22	−119.0 (3)
C20—C21—C22—Fe2	−59.8 (3)	C22—C23—Fe2—C19	−75.4 (3)
C21—C22—C23—C24	−0.1 (5)	C24—C23—Fe2—C19	165.6 (3)
Fe2—C22—C23—C24	59.8 (3)	C22—C23—Fe2—C20	81.5 (3)
C21—C22—C23—Fe2	−60.0 (3)	C24—C23—Fe2—C20	−37.4 (3)
C21—C20—C24—C23	0.2 (5)	C22—C23—Fe2—C21	38.1 (3)
Fe2—C20—C24—C23	−59.1 (3)	C24—C23—Fe2—C21	−80.9 (3)
C21—C20—C24—Fe2	59.3 (3)	C22—C23—Fe2—C18	−40.5 (6)
C22—C23—C24—C20	−0.1 (5)	C24—C23—Fe2—C18	−159.5 (5)
Fe2—C23—C24—C20	59.3 (3)	C22—C23—Fe2—C17	164.1 (4)
C22—C23—C24—Fe2	−59.4 (3)	C24—C23—Fe2—C17	45.1 (6)
C4—C5—Fe1—C1	−118.3 (4)	C22—C23—Fe2—C15	−118.1 (3)
C4—C5—Fe1—C2	−80.0 (3)	C24—C23—Fe2—C15	122.9 (3)
C1—C5—Fe1—C2	38.3 (2)	C22—C23—Fe2—C16	−160.9 (3)
C4—C5—Fe1—C10	164.4 (3)	C24—C23—Fe2—C16	80.1 (3)
C1—C5—Fe1—C10	−77.4 (3)	C22—C23—Fe2—C24	119.0 (3)
C4—C5—Fe1—C9	122.9 (3)	C24—C20—Fe2—C22	81.8 (3)
C1—C5—Fe1—C9	−118.9 (3)	C21—C20—Fe2—C22	−38.5 (3)
C4—C5—Fe1—C7	49.9 (6)	C24—C20—Fe2—C19	162.9 (4)
C1—C5—Fe1—C7	168.2 (5)	C21—C20—Fe2—C19	42.7 (6)
C4—C5—Fe1—C6	−165.1 (5)	C24—C20—Fe2—C23	38.1 (3)
C1—C5—Fe1—C6	−46.8 (7)	C21—C20—Fe2—C23	−82.1 (3)
C1—C5—Fe1—C4	118.3 (4)	C24—C20—Fe2—C21	120.2 (4)
C4—C5—Fe1—C8	80.9 (3)	C24—C20—Fe2—C18	−162.2 (3)
C1—C5—Fe1—C8	−160.8 (3)	C21—C20—Fe2—C18	77.6 (4)
C4—C5—Fe1—C3	−36.4 (3)	C24—C20—Fe2—C17	−120.2 (3)
C1—C5—Fe1—C3	81.9 (3)	C21—C20—Fe2—C17	119.6 (3)
C2—C1—Fe1—C5	118.4 (3)	C24—C20—Fe2—C15	−44.0 (7)
C11—C1—Fe1—C5	−120.4 (4)	C21—C20—Fe2—C15	−164.3 (5)
C5—C1—Fe1—C2	−118.4 (3)	C24—C20—Fe2—C16	−78.4 (4)
C11—C1—Fe1—C2	121.2 (5)	C21—C20—Fe2—C16	161.4 (3)
C2—C1—Fe1—C10	−119.9 (3)	C21—C20—Fe2—C24	−120.2 (4)
C5—C1—Fe1—C10	121.7 (3)	C20—C21—Fe2—C22	118.3 (5)
C11—C1—Fe1—C10	1.4 (4)	C20—C21—Fe2—C19	−161.8 (3)
C2—C1—Fe1—C9	−161.6 (3)	C22—C21—Fe2—C19	79.9 (4)
C5—C1—Fe1—C9	80.0 (3)	C20—C21—Fe2—C23	80.8 (3)
C11—C1—Fe1—C9	−40.4 (4)	C22—C21—Fe2—C23	−37.5 (3)
C2—C1—Fe1—C7	−47.9 (7)	C22—C21—Fe2—C20	−118.3 (5)
C5—C1—Fe1—C7	−166.3 (5)	C20—C21—Fe2—C18	−119.9 (3)
C11—C1—Fe1—C7	73.3 (7)	C22—C21—Fe2—C18	121.7 (3)
C2—C1—Fe1—C6	−78.3 (3)	C20—C21—Fe2—C17	−79.3 (4)
C5—C1—Fe1—C6	163.3 (3)	C22—C21—Fe2—C17	162.4 (3)
C11—C1—Fe1—C6	42.9 (4)	C20—C21—Fe2—C15	166.8 (4)
C2—C1—Fe1—C4	80.3 (3)	C22—C21—Fe2—C15	48.5 (7)
C5—C1—Fe1—C4	−38.2 (3)	C20—C21—Fe2—C16	−48.7 (8)
C11—C1—Fe1—C4	−158.5 (4)	C22—C21—Fe2—C16	−167.0 (5)
C2—C1—Fe1—C8	165.4 (5)	C20—C21—Fe2—C24	36.8 (3)
C5—C1—Fe1—C8	46.9 (6)	C22—C21—Fe2—C24	−81.5 (3)
C11—C1—Fe1—C8	−73.4 (6)	C17—C18—Fe2—C22	163.2 (3)
C2—C1—Fe1—C3	37.5 (2)	C19—C18—Fe2—C22	−77.0 (3)
C5—C1—Fe1—C3	−80.9 (3)	C17—C18—Fe2—C19	−119.8 (4)
C11—C1—Fe1—C3	158.7 (4)	C17—C18—Fe2—C23	−166.8 (4)
C3—C2—Fe1—C5	80.7 (3)	C19—C18—Fe2—C23	−47.0 (6)
C1—C2—Fe1—C5	−38.4 (2)	C17—C18—Fe2—C20	80.5 (3)
C3—C2—Fe1—C1	119.2 (4)	C19—C18—Fe2—C20	−159.7 (3)
C3—C2—Fe1—C10	−162.0 (3)	C17—C18—Fe2—C21	121.6 (3)
C1—C2—Fe1—C10	78.8 (3)	C19—C18—Fe2—C21	−118.6 (3)
C3—C2—Fe1—C9	165.3 (6)	C19—C18—Fe2—C17	119.8 (4)
C1—C2—Fe1—C9	46.1 (7)	C17—C18—Fe2—C15	−81.7 (3)
C3—C2—Fe1—C7	−78.3 (3)	C19—C18—Fe2—C15	38.2 (3)
C1—C2—Fe1—C7	162.6 (3)	C17—C18—Fe2—C16	−37.5 (3)
C3—C2—Fe1—C6	−119.2 (3)	C19—C18—Fe2—C16	82.4 (3)
C1—C2—Fe1—C6	121.6 (3)	C17—C18—Fe2—C24	49.7 (6)
C3—C2—Fe1—C4	36.7 (3)	C19—C18—Fe2—C24	169.5 (4)
C1—C2—Fe1—C4	−82.4 (3)	C18—C17—Fe2—C22	−42.6 (6)
C3—C2—Fe1—C8	−44.3 (7)	C16—C17—Fe2—C22	−162.4 (5)
C1—C2—Fe1—C8	−163.5 (6)	C18—C17—Fe2—C19	37.5 (3)
C1—C2—Fe1—C3	−119.2 (4)	C16—C17—Fe2—C19	−82.3 (3)
C9—C10—Fe1—C5	−77.1 (4)	C18—C17—Fe2—C23	167.1 (4)
C6—C10—Fe1—C5	165.3 (2)	C16—C17—Fe2—C23	47.3 (6)
C9—C10—Fe1—C1	−119.5 (4)	C18—C17—Fe2—C20	−118.0 (3)
C6—C10—Fe1—C1	123.0 (3)	C16—C17—Fe2—C20	122.3 (3)
C9—C10—Fe1—C2	−161.9 (3)	C18—C17—Fe2—C21	−76.2 (3)
C6—C10—Fe1—C2	80.5 (3)	C16—C17—Fe2—C21	164.1 (3)
C6—C10—Fe1—C9	−117.5 (4)	C16—C17—Fe2—C18	−119.7 (4)
C9—C10—Fe1—C7	80.6 (4)	C18—C17—Fe2—C15	81.7 (3)
C6—C10—Fe1—C7	−37.0 (3)	C16—C17—Fe2—C15	−38.1 (3)
C9—C10—Fe1—C6	117.5 (4)	C18—C17—Fe2—C16	119.7 (4)
C9—C10—Fe1—C4	−45.2 (7)	C18—C17—Fe2—C24	−159.5 (3)
C6—C10—Fe1—C4	−162.7 (5)	C16—C17—Fe2—C24	80.8 (3)
C9—C10—Fe1—C8	37.1 (3)	C19—C15—Fe2—C22	78.4 (3)
C6—C10—Fe1—C8	−80.5 (3)	C16—C15—Fe2—C22	−162.7 (3)
C9—C10—Fe1—C3	165.3 (5)	C14—C15—Fe2—C22	−42.6 (4)
C6—C10—Fe1—C3	47.7 (6)	C16—C15—Fe2—C19	118.9 (3)
C10—C9—Fe1—C5	121.7 (3)	C14—C15—Fe2—C19	−121.0 (5)
C8—C9—Fe1—C5	−118.2 (3)	C19—C15—Fe2—C23	120.7 (3)
C10—C9—Fe1—C1	78.7 (4)	C16—C15—Fe2—C23	−120.5 (3)
C8—C9—Fe1—C1	−161.1 (3)	C14—C15—Fe2—C23	−0.3 (4)
C10—C9—Fe1—C2	44.7 (8)	C19—C15—Fe2—C20	−164.1 (5)
C8—C9—Fe1—C2	164.9 (6)	C16—C15—Fe2—C20	−45.2 (7)
C8—C9—Fe1—C10	120.1 (5)	C14—C15—Fe2—C20	74.9 (7)
C10—C9—Fe1—C7	−81.9 (4)	C19—C15—Fe2—C21	43.1 (6)
C8—C9—Fe1—C7	38.2 (3)	C16—C15—Fe2—C21	161.9 (5)
C10—C9—Fe1—C6	−38.8 (3)	C14—C15—Fe2—C21	−78.0 (6)
C8—C9—Fe1—C6	81.4 (4)	C19—C15—Fe2—C18	−37.9 (3)
C10—C9—Fe1—C4	163.4 (3)	C16—C15—Fe2—C18	81.0 (3)
C8—C9—Fe1—C4	−76.5 (4)	C14—C15—Fe2—C18	−158.9 (4)
C10—C9—Fe1—C8	−120.1 (5)	C19—C15—Fe2—C17	−81.1 (3)
C10—C9—Fe1—C3	−164.0 (5)	C16—C15—Fe2—C17	37.7 (3)
C8—C9—Fe1—C3	−43.8 (8)	C14—C15—Fe2—C17	157.8 (4)
C6—C7—Fe1—C5	163.0 (4)	C19—C15—Fe2—C16	−118.9 (3)
C8—C7—Fe1—C5	42.9 (7)	C14—C15—Fe2—C16	120.1 (5)
C6—C7—Fe1—C1	−40.9 (7)	C19—C15—Fe2—C24	162.8 (3)
C8—C7—Fe1—C1	−161.0 (5)	C16—C15—Fe2—C24	−78.4 (3)
C6—C7—Fe1—C2	−77.0 (4)	C14—C15—Fe2—C24	41.8 (4)
C8—C7—Fe1—C2	162.8 (3)	C17—C16—Fe2—C22	162.6 (5)
C6—C7—Fe1—C10	37.9 (3)	C15—C16—Fe2—C22	43.7 (6)
C8—C7—Fe1—C10	−82.2 (3)	C17—C16—Fe2—C19	80.9 (3)
C6—C7—Fe1—C9	81.8 (3)	C15—C16—Fe2—C19	−38.0 (2)
C8—C7—Fe1—C9	−38.3 (3)	C17—C16—Fe2—C23	−161.8 (3)
C8—C7—Fe1—C6	−120.1 (5)	C15—C16—Fe2—C23	79.3 (3)
C6—C7—Fe1—C4	−160.7 (3)	C17—C16—Fe2—C20	−77.2 (3)
C8—C7—Fe1—C4	79.1 (4)	C15—C16—Fe2—C20	163.9 (3)
C6—C7—Fe1—C8	120.1 (5)	C17—C16—Fe2—C21	−41.2 (7)
C6—C7—Fe1—C3	−119.1 (3)	C15—C16—Fe2—C21	−160.1 (6)
C8—C7—Fe1—C3	120.7 (3)	C17—C16—Fe2—C18	37.1 (3)
C7—C6—Fe1—C5	−159.7 (5)	C15—C16—Fe2—C18	−81.9 (3)
C10—C6—Fe1—C5	−40.3 (7)	C15—C16—Fe2—C17	−118.9 (4)
C7—C6—Fe1—C1	164.7 (3)	C17—C16—Fe2—C15	118.9 (4)
C10—C6—Fe1—C1	−76.0 (3)	C17—C16—Fe2—C24	−119.0 (3)
C7—C6—Fe1—C2	122.0 (3)	C15—C16—Fe2—C24	122.1 (3)
C10—C6—Fe1—C2	−118.7 (3)	C20—C24—Fe2—C22	−81.3 (3)
C7—C6—Fe1—C10	−119.4 (4)	C23—C24—Fe2—C22	37.6 (3)
C7—C6—Fe1—C9	−80.9 (3)	C20—C24—Fe2—C19	−158.9 (5)
C10—C6—Fe1—C9	38.4 (3)	C23—C24—Fe2—C19	−40.0 (7)
C10—C6—Fe1—C7	119.4 (4)	C20—C24—Fe2—C23	−118.8 (4)
C7—C6—Fe1—C4	45.9 (6)	C23—C24—Fe2—C20	118.8 (4)
C10—C6—Fe1—C4	165.3 (4)	C20—C24—Fe2—C21	−37.0 (3)
C7—C6—Fe1—C8	−37.1 (3)	C23—C24—Fe2—C21	81.8 (3)
C10—C6—Fe1—C8	82.2 (3)	C20—C24—Fe2—C18	42.7 (7)
C7—C6—Fe1—C3	79.7 (4)	C23—C24—Fe2—C18	161.5 (5)
C10—C6—Fe1—C3	−161.0 (3)	C20—C24—Fe2—C17	78.6 (4)
C3—C4—Fe1—C5	−120.5 (4)	C23—C24—Fe2—C17	−162.6 (3)
C3—C4—Fe1—C1	−81.9 (3)	C20—C24—Fe2—C15	164.1 (3)
C5—C4—Fe1—C1	38.6 (2)	C23—C24—Fe2—C15	−77.1 (3)
C3—C4—Fe1—C2	−37.4 (3)	C20—C24—Fe2—C16	121.3 (3)
C5—C4—Fe1—C2	83.0 (3)	C23—C24—Fe2—C16	−119.8 (3)
C3—C4—Fe1—C10	−162.9 (5)		

### Hydrogen-bond geometry (Å, °)

**Table:**

*D*—H···*A*	*D*—H	H···*A*	*D*···*A*	*D*—H···*A*
C12—H12B···O1^i^	0.97	2.51	3.448 (5)	164
C13—H13B···O2^i^	0.97	2.55	3.400 (6)	147
C23—H23···O1	0.93	2.60	3.499 (5)	164
C10—H10···O2	0.93	2.58	3.457 (6)	157

Symmetry codes: (i) −*x*+1/2, *y*, *z*+1/2.
